# Fostering Lavender as a Source for Valuable Bioactives for Food and Pharmaceutical Applications through Extraction and Microencapsulation

**DOI:** 10.3390/molecules25215001

**Published:** 2020-10-28

**Authors:** Simona Daniela Radu (Lupoae), Liliana Mihalcea, Iuliana Aprodu, Sonia A. Socaci, Mihaela Cotârleț, Elena Enachi, Oana Crăciunescu, Vasilica Barbu, Anca Oancea, Francisc Vasile Dulf, Petru Alexe, Gabriela Elena Bahrim, Gabriela Râpeanu, Nicoleta Stănciuc

**Affiliations:** 1 Faculty of Food Science and Engineering, Dunărea de Jos University of Galati, Domnească Street 111, 800201 Galati, Romania; radu.daniela1308@yahoo.com (S.D.R.); Liliana.Mihalcea@ugal.ro (L.M.); Iuliana.Aprodu@ugal.ro (I.A.); Mihaela.Cotarlet@ugal.ro (M.C.); Elena.Ionita@ugal.ro (E.E.); Vasilica.Barbu@ugal.ro (V.B.); Petru.Alexe@ugal.ro (P.A.); Gabriela.Bahrim@ugal.ro (G.E.B.); Gabriela.Rapeanu@ugal.ro (G.R.); 2 Faculty of Food Science and Technology, Department of Food Science, University of Agricultural Sciences and Veterinary Medicine Cluj-Napoca, Calea Manastur 3-5, 400372 Cluj-Napoca, Romania; sonia.socaci@usamvcluj.ro; 3 National Institute of Research and & Development for Biological Sciences, 296 Splaiul Independentei, 060031 București, Romania; oana_craciunescu2009@yahoo.com (O.C.); oancea.anca@gmail.com (A.O.); 4 Faculty of Agriculture, Department of Environmental and Plant Protection, University of Agricultural Sciences and Veterinary Medicine Cluj-Napoca, Calea Manastur 3-5, 400372 Cluj-Napoca, Romania; francisc_dulf@yahoo.com

**Keywords:** lavender, extraction, supercritical fluids extraction, phytochemicals, microencapsulation, added-value, food flavoring

## Abstract

Lavender flowers were used in this study as a source of phytochemicals as naturally occurring antioxidants. Two different extraction techniques were applied, such as ultrasound-assisted (UAE) and supercritical fluids (SCE) methods. The comparative evaluation of the phytochemicals profile evidenced a higher content of chlorophyll *a* and *b* of 5.22 ± 0.12 mg/g dry weight (D.W.) and 2.95 ± 0.16 mg/g D.W, whereas the carotenoids content was 18.24 ± 0.04 mg/g D.W. in the SCE extract. Seven main compounds were found in both extracts: β-linalool, eucalyptol, linalool acetate, β-trans-ocimene, and limonene in SCE and linalool acetate, β-linalool, 6-methyl-2-(2-oxiranyl)-5-hepten-2-ol, linalool oxide, lavandulyl acetate and camphor in UAE. The (*n*-3) acids had a higher contribution in SCE. The extracts were microencapsulated in different combinations of wall materials based on polysaccharides and milk proteins. The four variants showed different phytochemical and morphological profiles, with a better encapsulating efficiency for proteins (up to 98%), but with a higher content of encapsulated carotenoids for polysaccharides, the latter showing remarkable antimicrobial activity against selected microorganisms. Carboxymethyl cellulose and whey proteins led to a double encapsulation of lipophilic compounds. The powders were tested in two food matrices as ingredients, with multiple targeted functions, such as flavoring, antimicrobial, antioxidant activity that can successfully replace synthetic additives.

## 1. Introduction

Our daily diets involve large quantities of many pigments, especially carotenoids, anthocyanins, and chlorophylls [1]. These compounds possess important biological functions in humans against major disorders like cancer, cardiovascular diseases, cataract, arteriosclerosis, macular degeneration, and other age-related diseases [2]. The health benefits of chlorophylls include anti-oxidant, anti-inflammatory, anti-bacterial, anti-carcinogenic, deodorizing, and wound healing activities [3]. Carotenoids offer beneficial effects in preventing some types of cancer, cardiovascular and degenerative diseases. Polyphenols, a class of low molecular weight secondary plant metabolites which includes flavonoids, exhibit excellent antioxidant properties. These compounds are well known for their antioxidant, antimicrobial, anti-carcinogenic activities, neuroprotective effects [4], and anti-inflammatory activities [5]. Therefore, the use of plant extracts as functional ingredients in various food, beverage and cosmetic applications is gaining growing interest. However, despite all these health benefits, there are limitations to the commercial-scale application of bioactives as natural colorants because they are very susceptible to environmental stresses exerted by oxygen, enzymes, light, high temperature, and acidic or alkaline pH, which result in degradation, oxidation, isomerization, and discoloration [6].

In recent years, the microencapsulation techniques aiming to develop a proper environment for different susceptible bioactives in a stable wall matrix as a mean of protecting functional compounds from processing conditions (like oxygen, pH, ionic strength, and temperature) and improving the bioavailability have gained significant interest. Microencapsulation allows creation of a physical barrier between the core and the wall materials, thus protecting the material from ambient conditions such as light, temperature, oxygen, humidity, and from interaction with other substances [7]. The key factors for an efficient microencapsulation are the type of wall material and the characteristics of compounds to be encapsulated, the microencapsulation techniques and process parameters [8]. 

Lavenders are aromatic evergreen shrubs from Lamiaceae family that have been traditionally used as culinary herbs and medicine for headaches, digestive troubles, burns, skin sores and insect bites [9]. *Lavandula* ssp. are well known and studied for the content in essential oils. For example, the essential oils from *L. stoechas* ssp. *luisieri* were analyzed and their antifungal, anti-inflammatory and antioxidant activities evidenced [10]. The essential oils from *L. pedunculata* were also analyzed and their antifungal activity demonstrated by Zuzarte et al. [11]. Teixeira et al. [12] suggested also that several extracts of both species showed antibacterial activity, anticholinesterase inhibition and antioxidant capacity.

The commercial interest for lavenders is due to the potential application in the fragrance industry (soaps, colognes, perfumes, skin lotion and other cosmetics), in aromatherapy (relaxant), in pharmaceutical preparations for its therapeutic effects as a sedative, spasmolytic, antiviral and antibacterial agent [13]. However, lavender has been employed in food manufacturing as natural flavoring for beverages, ice cream, candy, baked goods and chewing gum [14].

This study aimed to provide a scientific basis for using lavender as a source of bioactives in the food industry, with targeted functions, such as flavoring, antioxidants and antimicrobials, knowing that lavender is less exploited for its content in carotenoid or polyphenolic compounds. A preliminary step was employed, selecting two extraction techniques, such as a combined ultrasound assisted method, involving the use of the organic solvents, and supercritical CO_2_ fluids extraction, followed by the comparison of the profiles, thus targeting different bioactives, such as carotenoids, polyphenols, fatty acids and volatiles, from the perspectives of using extracts as oleoresins and/or nutraceuticals. Secondly, considering the well-known low stability of the bioactive compounds, the obtained extracts were microencapsulated in different combinations, using agar as basic encapsulant material and various adjuvants, such as carboxymethyl cellulose, acacia gum, whey protein and casein. Therefore, four experimental variants were obtained and characterized in terms of microencapsulation efficiency, phytochemical content (chlorophylls, carotenoids, polyphenols and flavonoids), antioxidant, cytotoxicity, and antimicrobial activities. The powders’ structure and morphology were analyzed by confocal laser microscopy. Further, the powders’ functionality was tested in two food products, namely ice cream and macaroons.

## 2. Materials and Methods

### 2.1. Reagents

Folin–Ciocalteu’s phenol reagent, sodium carbonate (Na_2_CO_3_), sodium nitrite (NaNO_2_), aluminum chloride (AlCl_3_), sodium hydroxide (NaOH), methanol, gallic acid, catechin, 1,1-diphenyl-2 picrylhydrazyl (DPPH), 6-Hydroxy-2,5,7,8-tetramethylchromane-2-carboxylic acid (Trolox), and the lipid standards and chemicals (used for oil extraction, fractionation, and preparation of fatty acid methyl esters (FAMEs)) were purchased from Sigma-Aldrich (Steinheim, Germany). Carbon dioxide with 99.99% purity was supplied by Messer S.A., Romania. For cell culture experiments, Minimum Essential Medium (MEM), fetal bovine serum (FBS), an L-glutamine, mixture of antibiotics (penicillin, streptomycin, neomycin: PSN) and Neutral red (NR) were purchased from Merck (Darmstadt, Germany). The NCTC (L cell, L-929, derivative of Strain L] (ATCC® CCL-1™) clone L929 cell line of mouse fibroblasts was from European Collection of Authenticated Cell Cultures (Sigma-Aldrich, Steinheim, Germany).

### 2.2. Extraction of Phytochemicals from Lavender 

The aerial parts of lavender flower (*Lavandula angustifolia*) were collected from natural populations occurring throughout the south-eastern regions of Romania (Galați, Romania), in July 2018. The plant material was air-dried at 40 °C and powdered. Two types of extractions were performed: one using organic solvents such as *n*-hexane and acetone (in ratio of 3:1, *v·v*^−1^) and ultrasonication (onwards called ultrasound assisted extract—UAE) and extraction using supercritical CO_2_ (onwards called SCE) to obtain extracts rich in essential oils and phytochemicals, such as volatiles, phenolics, chlorophylls and carotenoids.

### 2.3. Extraction of Phytochemicals from Lavender Using Ultrasound-Assisted Method

The phytochemicals were extracted from the grounded lavender by adding 10 mL of *n*-hexan:acetone (3:1, *v*·*v*^−1^) to 2 g of powder [15]. The mixture was homogenized using an Ultra-Turrax homogenizer (IKA Werke, Labortechnik, Staufen, Germany) for 2 min, followed by ultrasonication for 30 min. The ultrasonic bath (MRC Scientific Instruments, Holon, Israel) is equipped with a digital control system of sonication time, temperature and frequency. The extraction was performed at a constant frequency of 40 kHz, at a constant power of 100 W. Cold water was added to maintain a constant temperature (40 ± 1 °C) in the ultrasonic bath. After centrifugation at 8000× *g* for 10 min at 4 °C, the residue was re-extracted with 10 mL of *n*-hexan:acetone solutions (3:1, *v·v*^−1^) and the extraction was repeated four times. The supernatant was then concentrated under reduced pressure at 40 °C to dryness (AVC 2–18, Christ, UK). In order to be characterized in terms of phytochemicals and antioxidant activity, the extract was dissolved in a ratio of 1 mg·mL^−1^ in the extraction solvent and filtered through 0.45 µm membranes.

### 2.4. Supercritical Fluid CO_2_ Extraction

The pilot-plant extraction system (Natex, Prozesstechnologie GesmbH, Austria, Fabr. no. 10-023/2011) designed with a 2.0 L cylinder extraction vessel and two separators (S40 and S45) each with 1.5 L capacity were used for the supercritical CO_2_ extractions of the powdered lavender. Extraction was performed in multiple batches and the extractor basket was filled with ~0.400 kg of powdered lavender flowers for each extraction. During extraction, the CO_2_ was constantly chilled to remain liquid and recirculated. The solvent was brought to supercritical conditions at 7.38 MPa and a mass flow rate of 19.061 kg/h, as indicated by the data sheets from ABB software (ABB Mannheim, Germany). For all extractions the temperature in extraction vessel and the first separator (S40 for collection of heavy compounds) was set at 60 °C. Two extraction conditions were applied, 30 MPa and 40 MPa for 60 min and 120 min. In order to separate the fractions with different compositions, the pressure in the first separator (S40) was maintained at 15 MPa, while in the second separator (S45 that collected volatiles) decompression up to recirculation pressure of 5 MPa was set at the temperature of 25 °C [16]. At the end of each extraction, the decompression of the separators produced the extracts which were collected in dark bottles and kept under refrigeration temperature until further use in experiments. 

### 2.5. Phytochemicals Measurements

#### 2.5.1. Chlorophylls and Carotenoids Measurements

For estimating the chlorophyll and carotenoid contents, the absorbance was measured at 645 nm, 663 nm and 470 nm, using a spectrophotometer (Biochrom Libra S22 UV/Vis, Cambridge, United Kingdom). In order to calculate Chlorophyll *a*, Chlorophyll *b*, total Chlorophyll and total carotenoids, Arnon’s equations were used, as follow:

Chlorophyll *a* = 0.0127 × A_663_ − 0.00269 × A_645_ (1)

Chlorophyll *b* = 0.0229 × A_645_ − 0.00468 × A_663_ (2)

Total Chlorophyll = 0.0202 × A_645_ + 0.00802 × A_663_ (3)

Carotenoids = (1000 × A_470_ − 2.13 Chl *a* − 97.63 Chl *b*)/209 (4)

The results were expressed as mg per g of dry weight (mg/g D.W.) [16].

#### 2.5.2. Determination of Total Polyphenolic Content

The total phenol content (TPC) was determined using Folin–Ciocalteu reagent. In brief, 1 mL of Folin–Ciocâlteu reagent was added to 0.1 mL of ethanolic extract, followed by addition of 0.8 mL of sodium carbonate (6%) and 1 mL of distilled water. After incubation for 30 min, the absorbance was measured at 765 nm using a spectrophotometer (Biochrom Libra S22 UV/Vis, Cambridge, United Kingdom). Gallic acid was used as the standard and the results were expressed in mg of gallic acid equivalents (GAE) per g D.W. [16].

#### 2.5.3. Determination of Total Flavonoids Content

Total flavonoids content (TFC) was determined by adding 1 mL of distilled water and 0.075 mL of sodium nitrite (5%) to 0.25 mL of ethanolic extract solution. After incubation for 5 min in the dark at room temperature, 0.15 mL of AlCl_3_ 10%, 0.5 mL of sodium chloride (1 M) and 1 mL of distilled water was added and the mixture was further incubated for 10 min in the dark at room temperature. The absorbance was measured at 510 nm against a blank sample using a spectrophotometer (Biochrom Libra S22 UV/Vis, Cambridge, United Kingdom). Catechin was used as the standard and the results were expressed in mg of catechin equivalents (CE) per g D.W. [16].

#### 2.5.4. Fatty Acids Analysis

Fatty acid methyl esters (FAMEs) of the total lipids were derivatized by acid-catalyze transesterification using 1% sulphuric acid in methanol [17]. Lipids (1 mg) were re-suspended in 1 mL toluene in a Pyrex tube fitted with a condenser. Two milliliters of methanolic H_2_SO_4_ (1% *v*/*v*) were added, and the mixture was refluxed for 2 h at 80 °C. Water (5 mL) containing potassium chloride (5%; *w*·*v*^−1^) was added, and the transmethylated fatty acids extracted with hexane (2 × 5 mL) using Pasteur pipettes to separate the layers. The hexane layer was washed with water (4 mL) containing 2% potassium bicarbonate and dried over anhydrous sodium sulphate. Finally, the solution was filtered and the solvent was removed under reduced pressure in a rotary film evaporator. FAMEs were analyzed with a gas-chromatograph (GC) coupled with a mass spectrometer (MS), PerkinElmer Clarus 600 T GC-MS (PerkinElmer, Inc., Shelton, CT, USA) as described by [17]. The GC column was a Supelcowax 10 (60 m × 0.25 mm i.d., 0.25 µm film thickness; Supelco Inc., Bellefonte, PA, USA.). The oven temperature was set at 140 °C, then ramped to 220 °C at 7 °C/min, and held at 220 °C for 23 min. The injection volume was 0.5 µl (split ratio of 1:24) and the injector was set at 210 °C. Helium was used as the carrier gas with a constant flow rate of 0.8 mL·min^−1^. Mass spectra (E.I., positive ion electron impact mode) were recorded at 70 eV and using a trap current of 100 µA with a source temperature of 150 °C. The MS was scanned from *m*/*z* 22 to 395 for all GC-MS experiments. The FAMEs were identified by comparing their retention times with those of known standards (37 components FAME Mix, Supelco no. 47885-U) and the resulting mass spectra to those in our database (NIST MS Search 2.0). The concentration of each fatty acid was calculated as peak area percentage of total fatty acids. 

#### 2.5.5. Determination of Volatile Profile Using ITEX/GC-MS Technique

The extraction of volatile compounds from the lavender extracts was performed using in-tube extraction technique (ITEX) as described in our previous work [18] using 0.1 g of extract. The analysis of volatile compounds was carried out on a GCMS QP-2010 (Shimadzu Scientific Instruments, Kyoto, Japan) model gas chromatograph-mass spectrometer. The volatile compounds were separated on a Zebron ZB-5ms capillary column of 30 m × 0.25 mm i.d and 0.25 μm film thickness. The carrier gas was helium 1 mL·min^−1^ and the split ratio 1:100. The column oven temperature program was 50 °C (hold for 2 min) to 160 °C at 4 °C/min to 250 °C at 15 °C/min and maintained for 10 min. The injector, ion-source and interface temperatures were set at 250 °C. The MS detection used for this qualitative analysis was performed on a quadrupole mass spectrometer operating in full scan (40–500 *m*/*z*) electron impact (EI) at ionization energy of 70 eV. The volatile compounds were tentatively identified by first comparing the obtained mass spectra of each chromatographic peak to NIST27 and NIST147 mass spectra libraries (considering a minimum similarity of 85%) and then whenever possible by comparison with retention indices drawn from [19,20] (for columns with a similar stationary phase to the ZB-5ms column). This technique offers a qualitative assessment of volatile compounds, so the relative percentage of each compound was estimated as a fraction of its integrated ion area from total ion chromatograms (TIC) area (100%). 

### 2.6. Antioxidant Activity

The DPPH radical scavenging assay was conducted according to the method of Guo et al. [21] with slight modification. Briefly, 0.1 mL of extract was mixed with 3.9 mL of DPPH methanolic solution (1.5 × 10^−4^ M). The mixture was shaken vigorously and stored for 30 min in the dark at approximately 25 °C. The absorbance of the reaction mixture was then measured at 515 nm using a UV-Vis spectrophotometer (Biochrom Libra S22 UV/Vis, Cambridge, United Kingdom). The antioxidant activity was expressed as mMol Trolox/g D.W. using a calibration curve.

### 2.7. Microencapsulation of the Extracts

The two extracts were encapsulated in different polymers, as follows: the UAE dissolved in vegetable oil were encapsulated in two variants of agar (AA), carboxymethyl cellulose (CMC) and acacia gum (AG). The AA was mixed with CMC (variant 1) and AG (variant 2) at ratio of 1:1. The AA solutions with a total solid content of 2% (*w*·*w*^−1^) were prepared by mixing with homogenizer at 650 rpm for 3 h. In addition, CMC and AG, with a total solid content of 1% (*w*·*w*^−1^) were prepared through homogenization at 650 rpm for 6 h. Each variant was allowed to stand at 40 °C and 450 rpm for 30 min. 

Before microencapsulation, the S40 and S45 extracts were mixed. The SCE was microencapsulated in a combination of AA, whey protein isolate (WPI), whey protein hydrolysates (WPH) and casein (CN). AA was mixed with WPI and WPH (variant 3) and WPI and CN (variant 4) at ratio of 1:1. The AA solutions were prepared as described above. In addition, WPI, WHP and CN solutions, with a total solid content of 1% (*w*·*w*^−1^) were prepared by using homogenization at 650 rpm for 4 h. 

All coating solutions were kept overnight in the refrigerator at 4–8 °C, to obtain full hydration. An amount of 5.0 g extracts, weighed via precision balance, was dissolved in 20 mL vegetable oil and further added to the coating mixtures. In order to obtained capsules, the mixtures were homogenized by a high-speed homogenizer (IKA T25 digital Ultra-Turrax, Selangor, Malaysia) at 10,000 rpm for 10 min. The pH of the emulsions was brought to 3.75 using 0.1 N HCl to attain maximum complex coacervation. After this, the internal temperature of the reaction vessel was lowered to 4 ± 1 °C using ice water, followed by separation of coacervates. The samples were further frozen at −70 °C, and the ice crystals were then removed by freeze-drying (CHRIST Alpha 1–4 LD plus, Germany) at −42 °C under a pressure of 0.10 mBar for 48 h. Finally, the powders were collected and packed in metalized bags and stored in the freezer at −20 °C for later analyses. Each experiment was duplicated.

### 2.8. Microencapsulation Efficiency and Powders Characterization

The microencapsulation efficiency (ME) was estimated for all the compounds measured when characterizing the extracts, namely chlorophylls *a* and *b*, carotenoids, polyphenolics, and flavonoids. For the evaluation of the phytochemical in the powder, 200 mg of powder was accurately weighed and subjected to extraction with 10 mL of *n*-hexane:acetone (3:1, *w*·*v*^−1^). The mixture was homogenized using an Ultra-Turrax homogenizer (IKA Werke, Labortechnik, Staufen, Germany) for 5 min, followed by ultrasonication for 30 min at 40 ± 2 °C. After centrifugation at 8000× *g* for 10 min, the supernatant was used for measuring the phytochemicals concentration at selected wavelengths.

The concentration of above-mentioned compounds was used for estimating the encapsulation efficiency using the following formula (Equation (5)):

(5) $$ME\;\left(\%\right)=\frac{\left(C_{e}-C_{s}\right)}{C_{e}}\;\times \;100$$

where *ME*% corresponds to the microencapsulation efficiency, *C_i_* to the initial concentration of the bioactives in the extract and *C_f_* to the concentration of bioactives in the resulting supernatant.

### 2.9. Confocal Laser Scanning Microscopy 

A Zeiss LSM 710 confocal laser system was used for the investigations and the 3D images were captured and analyzed by the ZEN 2012 SP1 software (black edition). The technical specifications of the used equipment are: diode laser (405 nm), Ar-laser (458, 488, 514 nm), diode pumped solid state laser (561 nm) and HeNe-laser (633 nm), AxioObserver Z1 inverted microscope, 40× apochromatic objective (numerical aperture 1.4) and the FS49, FS38 and FS15 filters. To capture the autofluorescence of the native powders, the emission was measured at wavelength between 405–633 nm. In order to acquire the images, the microparticles were stained with two dyes: 4′,6-diamidino-2-phenylindole (1 μg/mL) and Red Congo (40 μM), in a ratio 3:1:1. To increase the signal-to-noise ratio, the frame average of eight scans was used.

### 2.10. Sizing the Particles of the Microencapsulated Powders

The powders resulted through freeze-drying were suspended in distilled water and homogenized for 60 min at 20 ± 1 °C using a magnetic stirrer. The particle size distributions were further determined by measuring the intensity of the light scattered by the laser beam, when passing through the complete hydrated sample. The particle size measurements were performed by means of the PA-200G Wet Laser Particle Size Analyser (MRC, Holon, Israel). The dispersing medium used for particle size measurements was distilled water. The particle size was expressed both as the surface area mean (D [2,3] or Sauter Mean Diameter) and the volume moment mean (D [3,4] or De Brouckere Mean Diameter). Moreover, particle size distribution was presented as maximum diameter bellow which 10% (D10), 50% (D50) and 90% (D90) of the samples volume exist.

### 2.11. Antimicrobial Activity

The method described by Cortes-Zavaleta et al. [22] was used to analyze the antimicrobial activity against three spoilage microorganisms, such as: *Aspergillus niger* MIUG M5, *Penicillium expansum* MIUG M11 and *Bacillus subtilis* MIUG B1 that belong to the Microbial Collection of Dunarea de Jos University, Galați, Romania. An amount of 0.5 g of each variant was homogenized with 45 mL of sterile potato dextrose agar (PDA) or plate count agar (PCA) medium, cooled down at 42 °C and poured into Petri dishes. The plates were centrally inoculated with 5 μL spore suspension with a final concentration of 1 × 10^6^ spores/mL and incubated at 25 °C for 5 days (for molds) and at 37 °C for 48 h (for bacteria). Control plates containing PDA and PCA media mixed with sterile distillate water in the same proportions as above were also prepared and inoculated. After the incubation period, the area of the growth in both treated (A_T_) and control (A_C_) plates were determined from the mean perpendicular diameter measurements assuming a circular growth. The inhibition ratio (IR) was calculated using the following formula (6):

(6) $$IR\;\left(\%\right)\;=\;\frac{A_{C}\;\;-\;\;A_{T}}{A_{C}}\times 100$$

### 2.12. Cell Culture and Treatment

For cell culture experiments, a mouse fibroblasts cell line (NCTC clone L929, ECACC, Sigma-Aldrich, Germany) was used. Cells were cultured in Minimum Essential Medium (MEM) supplemented with 10% (*v·v*^−1^) fetal calf serum (FCS), 2 mM L-glutamine and 1% (*v·v*^−1^) antibiotic mixture (penicillin–streptomycin–neomycin), in a humidified atmosphere with 5% CO_2_, at 37 °C, until subconfluence. Trypsinized cells were seeded in 96-well microplates, at a density of 4 × 10^4^ cells/mL and allowed to adhere by incubation at 37 °C, in humidified atmosphere with 5% CO_2_, for 24 h. Then, the culture medium was replaced with fresh medium containing different concentrations of sterilized samples ranging from 10–1000 μg/mL. The plates were incubated at 37 °C under standard conditions, for 24 h and 48 h, respectively. Cells cultivated in normal culture medium were used as negative control culture.

### 2.13. In Vitro Cytotoxicity Test

Cell viability in treated cultures was evaluated by Neutral red (NR) assay, as previously described [23]. Briefly, at the end of each incubation period, the culture medium was removed from each well and the cells were incubated with 50 μg/mL NR solution, at 37 °C, for 3 h. After cell washing, the incorporated dye was released in 1% (*v*·*v*^−1^) acetic acid solution in 50% (*v*·*v*^−1^) ethanol by gentle shaking, for 15 min. The amount of uptaken dye was directly proportional to the number of viable cells. The optical density was measured at 540 nm in a Sunrise microplate reader (Tecan, Austria). The results were reported as percentage relative to the control culture, considered 100% viable.

### 2.14. Cell Morphology

After 48 h of cultivation in the presence of samples, cell morphology was observed in images acquired using a phase-contrast inverted microscope Axio Observer D1 equipped with digital camera (Carl Zeiss, Germany).

### 2.15. Testing the Microencapsulated Powder in Food Matrices

The ice cream was prepared using a classic recipe, where variants 1 and 2 were added in milk as an ingredient. The recipe involved the use of condensed milk, cream, sugar, and microencapsulated powder in ratio of 1%. The ice cream recipe involved a mixing step (5 min) of milk with sugar and variants 1 and 2. The cream was obtained by foaming, after which all the ingredients were mixed. The ice cream mix was introduced at −18 °C for 6 h, with homogenization in the first 2 h at half-hourly intervals.

For the macaroons, the recipe involved the use of the walnuts, almonds, sugar, egg whites and variants 3 and 4 in ratio of 1%. The first stage consisted in crushing the nuts and almonds. The egg whites were mixed with sugar until a stable foam was obtained. All components were mixed well together with variants 3 and 4, after which the macaroons were formed, each weighting of 20 g. The macaroons were obtained by heat treatment at 175 °C for 15 min in a convection oven. For each type of product, a control sample was obtained. The ice cream samples were coded I_M_, I_1_ and I_2_, whereas macaroons were coded as M_M_, M_3_ and M_4_, respectively.

### 2.16. Statistical Analysis of Data

All experimental measurements were performed at least in triplicate, and the results are presented as mean value ± standard deviation (SD). One-way analysis of variance (ANOVA) and Tukey’s test with a 95% confidence interval was applied using Minitab 18 software to identify significant differences among results. Statistical analysis of the cell culture results was performed using two-tailed, two-sample equal variance Student’s *t*-test. Differences were considered statistically significant at *p* ≤ 0.05 and *p* ≤ 0.01 as minimal levels of significance.

## 3. Results and Discussion

### 3.1. Total Phytochemical Characterization of the Lavender Flowers Extracts

In our study, two extracts were obtained with different total phytochemicals profiles, thus aligning with numerous screening studies of various plant materials that have been performed in order to find naturally occurring antioxidants to be used in food or medicinal preparations, as replacements for potentially harmful synthetic additives [24]. Therefore, the two fractions resulting from SCE were mixed and characterized in terms of chlorophylls, carotenoids, polyphenolics, flavonoids and antioxidant activity. For volatile and fatty acids, the two fractions were analyzed separately. The UAE showed a chlorophyll *a* and *b* content of 2.10 ± 0.08 mg/g D.W and 0.69 ± 0.02 mg/g D.W, respectively, whereas the content in total carotenoids was 0.42 ± 0.01 mg/g D.W. The SCE displayed higher chlorophyll *a* and *b* content of 5.22 ± 0.12 mg/g D.W. and 2.95 ± 0.16 mg/g D.W, whereas the carotenoids content was 18.24 ± 0.04 mg/g D.W.

The TPC had values of 23.61 ± 0.75 mg GAE/g D.W. in UAE and almost four times more in SCE (80.95 ± 0.67 mg GAE/g D.W.). The same trend was observed in TFC, with values of 19.45 ± 0.45 mg CE/g D.W. and 34.02 ± 0.51 mg CE/g D.W., respectively.

The GC-MS analysis of the extracts led to the identification of 44 and 26 volatile compounds in SCE and UAE, respectively. Table 1 shows the main chemical composition of the extracts, revealing the presence of seven main compounds in SCE (β-linalool, eucalyptol, linalool acetate, β-trans-ocimene, and limonene) (Figure S1 in Supplementary Materials), whereas in UAE the main chemical showed the presence of seven main compounds (linalool acetate, β-linalool, 6-methyl-2-(2-oxiranyl)-5-hepten-2-ol, linalool oxide, lavandulyl acetate and camphor).

In both extracts, the main volatile compounds represented more than 71% of the extract composition. No differences in volatile profiles were found between S40 and S45 fractions, but significant differences occurred between the two types of extracts, SCE and UAE, respectively. Although present in small proportions, SCE fractions are richer in volatile compounds than the UAE. For example, the S40 and S45 fractions contained α-pinene, 3-heptanone, 6-methyl-, camphene, sabinene, β-pinene, 1-Octen-3-ol etc. However, the UAE contained about two times higher quantities of β-myrcene, 6-Methyl-2-(2-oxiranyl)-5-hepten-2-ol, linalool oxide (fr.1) and octen-1-ol, acetate and a significant lower proportion of camphor. Significant differences were found in limonene, eucalyptol, β-trans-ocimene, β-linalool proportions, with a higher proportion in S45 and less in UAE. Regarding the proportions of linalool acetate, the UAE contained the highest amount (25.86 ± 1.24%), followed by S40 fraction (18.08 ± 0.87%) and S45 (15.28 ± 1.25%).

The chemical composition of lavender and lavandin essential oils is characterized by the presence of terpenes (e.g., linalool and linalyl acetate) and terpenoids (e.g., 1,8-cineole), which are mainly responsible for the characteristic flavor and the biological and therapeutic properties [25].

The effects of three operating conditions of supercritical CO_2_ extraction, namely pressure, temperature and time, on yield, chemical composition and antioxidant activity of lavender essential oil were investigated by Danh et al. [16]. These authors suggested that four major compounds were detected in all extracts, namely linalool (∼43%), linalyl acetate (∼23%), camphor (∼8%) and borneol (∼6.6%), which combined made up about 80% of the oil yields.

Da Porto et al. [14] used three methods, such as hydrodistillation, SCE and UAE to comparatively evaluate the flavor compound from *Lavandula angustifolia* L. extracts. These authors suggested linalool and linalyl acetate as the principal components in the extracts, with higher concentrations of linalool, camphor, linalyl acetate and (E)-caryophyllene in SCE extracts. 

Figure S2 in Supplementary Materials shows the typical GC-MS chromatogram, which reveals the presence of seven peaks in SCE (Figure S2a) and eight peaks in UAE (Figure S2b), respectively. The type and ratio of fatty acids identified in the lavender extracts are shown in Table 2.

The fatty acid profile highly depends on the type of extraction and SCE fraction. From Table 2, it can be seen that the S40 fraction presented the highest proportions of α-linolenic (31.67 ± 1.58%) and palmitic acids (49.80 ± 2.31%), whereas UAE showed the highest proportion of linoleic (19.66 ± 0.85%), oleic (27.21 ± 1.10%) and stearic acids (13.73 ± 0.62%).

It can be observed that the all extracts accumulate a relative high concentration of polyunsaturated fatty acids (with the highest value of 42.43% in S45) and lower concentrations of monounsaturated fatty acids (with the highest value of 27.88 ± 1.20% in UAE and the lowest of 5.93 ± 1.20% in S40). The *n*-3 acids had a higher contribution in S40 (31.67 ± 1.59%) compared to *n*-6 acids (7.66 ± 1.58%) and lower in UAE, of 11.82 ± 0.55% and 19.66 ± 0.90%, respectively. Therefore, based on our results, it can be concluded that the extraction method highly influenced the phytochemical profile of the extracts. Thus, only with the exact knowledge of the phytochemical profile and related biological activities, it will be possible to develop a new generation of standardized, multifunctional plant-based formula, fulfilling actual standards for quality, safety and efficiency.

### 3.2. Microencapsulation Efficiency

Usually, health-promoting bioactives, such as carotenoids, polyphenols, polyunsaturated fatty acids etc. are prone to degradation, due to their chemical structure and processing and storage conditions. Encapsulation is a technique that preserves the physicochemical and health-promoting properties associated with bioactive compounds [26], thus promoting protection against environmental conditions and allowing controlled release in target sorption areas. The choice of suitable biopolymers as wall materials is critical to the success of microencapsulation, because the type of wall material is decisive for the physicochemical and morphological properties of the produced powders, affecting the encapsulation efficiency, shelf-life, and the degree of protection of the sensitive core materials [6]. Four variants were tested in our study, by combining agar with two polysaccharides and two proteins. The microencapsulation efficiencies were evaluated for each tested compound and are given in Table 3.

From Table 3 it can be seen that the use of proteins as microencapsulation adjuvants along with agar resulted in higher ME for each phytochemical. For example, for chlorophyll *a,* the ME was 70.95 ± 1.45%, in variant one, 68.57 ± 2.47% in variant two, 96.62 ± 1.84% and 98.01 ± 2.51% in variants three and four, respectively. Variants three and four showed the highest ME values for all the phytochemicals, suggesting that whey proteins and caseins are efficient wall materials.

Kang et al. [6] encapsulated unstable chlorophylls using different blends of gum Arabic (GA) and maltodextrin (MD) (GA–MD ratios of 5:5, 3:7, and 0:10) by spray-drying. These authors reported that an increase in concentration of MD in the wall materials was associated with lower moisture content (0.56%), higher encapsulation efficiency (77.19%), chlorophyll content (46.78 µg/g dry powder), degree of crystallinity, and thermal stability of microcapsules.

### 3.3. Powders Characterization

The phytochemical profile of the powders is given in Table 4. A significant different pattern was found for all variants, with higher contents of chlorophylls and carotenoids in variants one and two. Polyphenolics and flavonoids were found in higher concentration in variants three and four, leading to higher antioxidant activity. Kang et al. [6] reported that the chlorophyll content significantly increased with an increase in maltodextrin concentration in wall materials, and varied between 34.77 ± 0.12 µg/D.W. powder and 46.78 ± 0.14 µg/D.W powder for gum acacia: maltodextrin ratio of 5:5 and 0:10, respectively.

From the data presented in Table 4, it can be concluded that phytochemical profile of the freeze-dried microencapsulated powders highly depends on the phytochemical profile of the extract and wall material. Therefore, the combination of AA with CMC and AG was more effective in retention of carotenoids, whereas combining WPI with WPH and CN led to a higher retention of polyphenols. When comparing WPH and CN, from Table 4 it can be observed that WPH and WPI were more efficient in carotenoid retention.

### 3.4. Morphology and Particle Size of the Microencapsulated Powders

**Figure 1:**
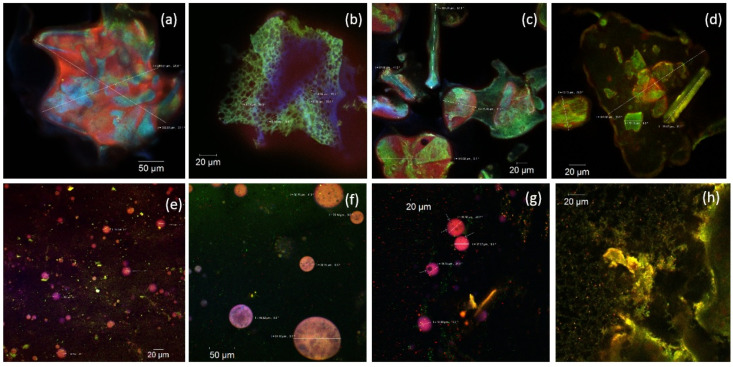
Confocal laser scanning microscopy images of the native variant 1 (**a**), variant 2 (**b**), variant 3 (**c**), variant 4 (**d**) microencapsulated powders, and of the stained variant 1 (**e**), variant 2 (**f**), variant 3 (**g**), variant 4 (**h**) microencapsulated powders.

In the native samples obtained through the novel microencapsulating formulas (Figure 1a–d), thin scales with irregular, asymmetric forms were observed, their emission being throughout the whole spectral range due to their complex composition. Nonetheless, the variants one and two seem to have a more uniform distribution of the biologically active compounds inside the microencapsulating biopolymeric matrices. 

It is well-known that lavender contains besides lipophilic components (essential oils with high concentrations of alcohols, aldehydes, esters, ketones and sesquiterpenes), hydrophilic compounds (phenolic compounds, flavonoids—mainly flavone glycosides, anthocyanins, tannins, etc.) that are responsible for the anti-inflammatory, antibacterial, antifungal, sedative and spasmolytic activity [27]. The particles sizes and their morphology depend on the microencapsulation method and on the component’s ratio in the microencapsulation matrix formula. Large scale formations with the average size between 40 and 80 µm (Figure 1), with irregular outline and porous structures (like micro-cavities or fissures with 6–8 µm diameters like in Figure 1b) were visualized. Some of them are very large (244.57–306.93 µm) such as the ones seen in Figure 1a. The dyed microencapsulated powders revealed the presence of lipophilic compounds in the form of spheroids with different diameters ranging in average between 10 and 30 microns (Figure 1e–h). In variant four, the spheres presented the smallest values, most of them having a diameter smaller than 2 µm (Figure 1d). In the other variants, 20% of the spherosomes had diameters of more than 60 µm (Figure 1b). Moreover, within these giant spherosomes one can observe microvesicles (the arrows in Figure 1b,c), suggesting a possible double encapsulation. The various protein components used in the microencapsulation process usually emit in a wide domain of wavelengths ranging from 550 to 650 nm. They formed a complex matrix in which the lavender’s biologically active compounds were encapsulated. Therefore, it can be concluded that CMC (Figure 1f) and WPH (Figure 1g) used for the microencapsulation led to a double encapsulation of the lipophilic compounds with biological value from the lavender extract, although the finest and the most homogeneous powders were obtained when AG (Figure 1e) and CN (Figure 1h) matrices were used.

Knowledge of the particle properties of the microencapsulated powder is important for better understanding and tailoring the quality of the final product. Therefore, in order to distinguish between coarse and fine particulates present in the samples, both D [2,3] and D [3,4] were measured. The D [2,3] is mainly useful for monitoring the presence of fine particles in the mixture, whereas D [3,4] provides a good estimation of the size of the particles representing the majority of the sample volume. Analyzing the results presented in Table 5, one can see that for all microencapsulated powders the size of the coarser particles is slightly higher compared to the fines. 

The D [2,3] values decrease in the following order variant 4 > variant 2 > variant 3 > variant 1 (Table 5). The same trend was observed for the size of the coarse particles present in the microencapsulated powders, D [3,4] values ranging from 4.684 µm registered for variant one and 5.108 µm for variant four. The largest variation of the particle size was registered in case of variant four, when 80% of sample volume had particle diameters ranging from 3.654 to 7.860 µm. On the other hand, the most uniform microencapsulated powder in terms of particle size was variant one with the narrowest D10–D90 range of 3.613–5.698 µm.

### 3.5. Antimicrobial Activity

The highest antimicrobial activity of 100% was found for variants one and two against *Penicillium expansum* MIUG M11 and *Bacillus subtilis* MIUG B1. The most resistant fungal strain turned out to be *Aspergillus niger* MIUG M5 for all four variants, showing no or lower antifungal activity (0 up to 6.52%). The higher antimicrobial activity of variants one and two may be associated with the higher contents of carotenoids, and probably free fatty acids. There are numerous studies reporting the antimicrobial potential of carotenoids on different spoilage and pathogen microorganisms. Most probably, the mechanism of antimicrobial activity involves the precipitation of membrane proteins, resulting in microbial cell lysis. However, given the inhibition ratio results, the biophysical and biochemical effects of fatty acids on the molecular structure of the cytoplasmic membrane might have potential. The hydrocarbon chain of added long-chain fatty acids might be inserted into the phospholipid bilayer of the membrane, thus increasing the destabilization of the membrane, as explained by Kitahara et al. [28]. The higher antimicrobial activities of the variants one and two may be a synergistic result of the fatty and carotenoid content. These results are in agreement with Czerwińska and Szparaga [29], who reported the antimicrobial activity of some aqueous extracts of *Lavandula vera* L. against *Bacillus subtilis*, *Aspergillus glaucus* and *Aspergillus niger*. *Bacillus subtilis* and *Aspergillus niger* were the most resistant strains. 

In conclusion, the significant antimicrobial effect of variants one and two against selected strains of molds and bacteria may be attributed to the dominant presence of carotenoids in the UAE samples, in addition to the complexity of the fatty acid’s compositions and the microbial skeletons characteristics [30]. Moreover, the existence of unsaturated fatty acids in the both types of extracts has a powerful effect on the antimicrobial activity due to increased cell membrane permeability [31]. Nevertheless, our results showed noticeable antibacterial effect mainly due to the presence of carotenoids and chlorophyll.

### 3.6. In Vitro Cytotoxicity

**Figure 2:**
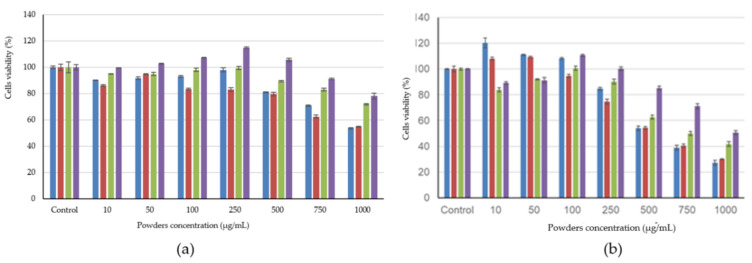
Cell viability on L929 fibroblasts cultured in the presence of microencapsulated powders of lavender extracts. (Variant 1 blue, variant 2 red, variant 3 green and variant 4 purple) after 24 h (**a**) and 48 h (**b**). The results were expressed as a relative percentage to the untreated control sample, considered 100% viable. The values represent mean values ± SD (*n* = 3), * *p* < 0.05 compared to the control sample.

The samples’ cytotoxicity was evaluated in L929 fibroblast cell culture by NR assay. The results obtained for cell viability at 24 h (a) and 48 h (b) of cultivation are presented in Figure 2. 

It can be seen that variants one and two had a similar behavior; after 24 h of culture, variants 1 and two were cytocompatible in the concentration range 10–500 µg/mL, and the values for cell viability varied between 80 and 98%. At higher concentrations, fibroblast viability decreased to 54%, indicating moderate cytotoxicity. After 48 h, variants one and two stimulated cell proliferation in the concentration range of 10–100 µg/mL and 10–50 µg/mL, respectively. Values for cell viability were significantly higher than those for control (*p* < 0.05), ranging from 108 to 120%. This behavior can be correlated with the release of biologically active compounds from encapsulated matrices.

Microencapsulated variant three was cytocompatible in the concentration range 10–750 µg/mL after 24 h of culture (83–99% cell viability) and in the range of 10–250 µg/mL after 48 h (90–101% cell viability). At higher concentrations, cell viability decreased to 42% after 48 h. The encapsulation matrix may not have allowed the release of biologically active compounds or the amount released has not sufficiently stimulated cell viability.

After 24 h of cultivation, variant four was cytocompatible throughout the tested concentration range of 10–1000 µg/mL, but after 48 h of culture, the cytocompatibility interval decreased to 10–500 µg/mL. Concentrations of 750–1000 µg/mL showed moderate cytotoxicity after 48 h of cultivation (50–70% cell viability). In addition, variant four stimulated cell proliferation at concentrations between 100and 500 µg/mL even after 24 h of culture and at concentrations of 100 µg/mL after 48 h of culture. These results indicate a rapid release of biologically active compounds, which exerted a stimulating effect on cellular metabolism.

Zhang et al. [32] used an in vitro test on six different cells to evaluate the cytotoxicity of the citron extracts, showing that cytotoxicity increased in some cell lines, like hCPCs, hEPCs, and H9C2, after treatment with peptide and liposome based citron nanoparticles. However, the citron–peptide complex caused genotoxicity in the NIH-3T3 and H9C2 cell lines, but not cytotoxicity.

Aprodu et al. [33] also studied the effect of black rice anthocyanins microencapsulated in milk proteins and peptides as possible toxic compounds on L929 fibroblast cell culture, suggesting that treatment with higher concentrations of microencapsulated powders (750–1000 μg/mL) induced a decrease in cell viability, but the values were higher than 80%, indicating non-cytotoxicity.

### 3.7. Value Added Food Products

The powders were added as ingredients in two products, namely ice cream and macaroons. The variants one and two were added in ice cream, whereas variants three and four were added in macaroons in proportions of 1%. The products were tested for antioxidant activity in order to demonstrate the added value. The antioxidant activities of the ice cream samples were 1.22 ± 0.04 mMol Trolox/g D.W. for I_M_, 1.54 ± 0.05 mMol Trolox/g D.W. for I_1_, and 1.65 ± 0.02 mMol Trolox/g D.W. for I_2_. It can be seen that the antioxidant activity was higher in the products with added powders, with approx. 26% and 35% in I_1_ and I_2_, respectively.

The macaroons showed rather high antioxidant activity, of 9.40 ± 0.49 mMol Trolox/g D.W. for M_M_, 9.73 ± 0.04 mMol Trolox/g D.W. for M_3_ and 11.28 ± 1.08 mMol Trolox/g D.W. for M_4_, respectively. In this case, the increase in antioxidant activity was with only 3.5% in case of M_3_ and with 20% in case of M_4._ Some important aspects should be considered in choosing the food matrix to which these functional ingredients are added, and these are the possible interactions between components and the thermal processing, that may lead to the degradation of biologically active compounds. This may explain why in ice cream samples, the added value is higher compared to the macaroons, which underwent a baking step.

## 4. Conclusions

The actual trend in developing natural ingredients with diverse biological functionalities, such as flavoring, antimicrobial and antioxidant activities, thus providing potential health benefits was exploring in our study. Lavender is a rich source of valuable volatiles, carotenoids, chlorophylls, polyphenols, flavonoids with significant biological activities and application on therapy. Two selected extraction methods were comparatively analyzed, based on phytochemical content, namely supercritical fluids and ultrasound assisted extractions. The applied extraction techniques were different in approach, methodology and costs, involving the use of the organic solvents assisted by ultrasound and extraction with supercritical fluids (CO_2_ supercritical). The obtained results evidence both quantitative and qualitative differences among the extracts. The richest extracts, in terms of amount of isolated compounds, were those obtained with supercritical fluids extraction. The extract processed through supercritical fluids had a wider spectrum of volatile compounds and fatty acids, as well as significantly higher contents of chlorophylls, carotenoids and polyphenols when compared with ultrasound-assisted extracts. Although the supercritical method allows effective and quick extraction, operates with moderate temperatures, eliminates clean-up steps, avoids the use of harmful organic solvents, it requires significant investments in equipment and maintenance.

When extracted, the biological and technological applications of bioactives can be compromised, as they are generally sensitive to oxidation and high temperatures, which lead to marked discoloration during processing and storage. Therefore, complex coarcevation and freeze-drying were applied to obtain stable powders, based on different polymers’ combinations. The powders were characterized for total phytochemical and morphological profiles, with a better encapsulating efficiency for proteins, but with a higher content of encapsulated carotenoids for polysaccharides, the latter showing remarkable antimicrobial activity.

Carboxymethyl cellulose and whey proteins led to a double encapsulation of the lipophilic compounds from the lavender extract, although the finest and the most homogeneous powders were obtained when acacia gum and casein were used. The selected variants showed a cytocompatible behavior in certain concentration, whereas some proliferation of cells were highlighted due to the release of the bioactives from microcapsules.

Significant knowledge was provided by the current study from the perspectives of developing ingredients, with targeted multiple functions, such as flavoring, antimicrobial, antioxidant activity that can successfully replace synthetic additives and developing value-added food products.

Currently, our studies continue to evaluate the flavor and fatty acids profile of microencapsulated powders and microencapsulation efficiency of the selected compounds.

## Figures and Tables

**Table 1:** Volatile compounds in lavender of lavender extracts.

Compound Name	% of Total Peak Areas		
	SCE (S40)	SCE (S45)	UAE
2-Pentanone, 4-hydroxy-4-methyl-	-	-	9.24 ± 0.89 *
2(5*H*)-Furanone, 5.5-dimethyl-	-	-	0.37 ± 0.10
1-Hexanol	0.05 ± 0.01	0.08 ± 0.01	-
Tricyclo[2.2.1.0(2,6)]heptane, 1,7,7-trimethyl-	0.03 ± 0.01	0.13 ± 0.02	-
Origanene	0.06 ± 0.01	0.15 ± 0.02	-
α-Pinene	0.30 ± 0.02	0.77 ± 0.04	-
3-Heptanone, 6-methyl-	0.11 ± 0.05	0.11 ± 0.01	-
Heptane, 2.5.5-trimethyl-			0.24 ± 0.17
Camphene	0.77 ± 0.09	1.97 ± 0.11	-
u.i.	0.17 ± 0.02	0.86 ± 0.11	-
2-Thujene	0.04 ± 0.01	0.08 ± 0.01	-
4(10)-Thujene (Sabinen)	0.10 ± 0.01	0.47 ± 0.02	-
β-Pinene	0.25 ± 0.05	0.41 ± 0.03	-
1-Octen-3-ol	0.20 ± 0.05	0.17 ± 0.01	-
3-Octanone	1.30 ± 0.07	1.09 ± 0.05	0.30 ± 0.10
β-Myrcene	1.34 ± 0.10	1.32 ± 0.11	2.83 ± 0.45
Butanoic acid, butyl ester	0.15 ± 0.01	0.18 ± 0.01	-
u.i.	0.09 ± 0.01	0.10 ± 0.01	-
Acetic acid, hexyl ester (1-Hexyl acetate)	0.85 ± 0.08	0.80 ± 0.11	-
α.-Terpinene	0.08 ± 0.01	0.11 ± 0.01	-
*p*-Cymene	0.83 ± 0.10	1.16 ± 0.41	0.23 ± 0.01
Limonene	3.48 ± 0.54	4.87 ± 0.58	0.97 ± 0.15
Eucalyptol	16.72 ± 0.98	17.62 ± 1.20	2.10 ± 0.08
β-*trans*-Ocimene	4.02 ± 0.45	5.13 ± 0.74	1.56 ± 0.02
β-*cis*-Ocimene	1.58 ± 0.23	1.60 ± 0.12	1.48 ± 0.09
γ-Terpinene	0.16 ± 0.08	0.36 ± 0.11	-
6-Methyl-2-(2-oxiranyl)-5-hepten-2-ol	3.98 ± 0.23	3.45 ± 0.41	6.74 ± 1.02
Linalool oxide (fr.1)	3.04 ± 0.25	2.66 ± 0.52	5.76 ± 0.28
β-Linalool	19.94 ± 1.01	20.44 ± 1.20	15.18 ± 1.20
Octen-1-ol, acetate	1.01 ± 0.05	0.97 ± 0.11	1.85 ± 0.07
2,4,6-Octatriene, 2,6-dimethyl-, (E, Z)-	0.71 ± 0.11	0.58 ± 0.15	0.18 ± 0.02
Camphor	10.3 ± 0.74	7.82 ± 1.21	4.39 ± 0.23
Lavandulol	0.35 ± 0.11	0.33 ± 0.08	-
Borneol	1.76 ± 0.25	2.38 ± 0.54	3.16 ± 0.78
1-Terpinen-4-ol	1.41 ± 0.47	1.42 ± 0.69	1.46 ± 0.03
u.i.			1.30 ± 0.04
Butanoic acid, hexyl ester	-	0.37	-
*n*-Hexyl butanoate	0.44 ± 0.11	-	-
α-Terpineol	-	0.20 ± 0.01	-
Isobornyl formate	-	0.11 ± 0.01	0.22 ± 0.01
Linalool acetate	18.08 ± 0.87	15.28 ± 1.25	25.86 ± 1.24
Lavandulyl acetate	2.63 ± 0.56	2.13 ± 0.52	5.62 ± 0.78
u.i.	0.15 ± 0.08	0.08 ± 0.01	1.06 ± 0.06
8-Hydroxylinalool	-	-	2.54 ± 0.24
Nerol	0.14 ± 0.09	0.11 ± 0.01	0.53 ± 0.08
u.i. **	0.38 ± 0.11	0.24 ± 0.05	1.17 ± 0.09
Caryophyllene	1.73 ± 0.12	1.20 ± 0.02	1.86 ± 0.08
α-Bergamotene	0.08 ± 0.01	0.05 ± 0.01	-
β-Farnesene	0.62 ± 0.22	0.49 ± 0.01	0.43 ± 0.09
γ-Muurolene	0.09 ± 0.01	-	-
u.i.	0.15 ± 0.04	0.17 ± 0.01	0.98 ± 0.14
Acetic acid. hexyl ester	-	-	0.39 ± 0.17

*—standard deviation, ** unidentified., SCE–supercritical fluid extracts obtained in separator S40 and 45, respectively, UAE–ultrasound-assisted extract.

**Table 2:** Fatty acid composition (% of total fatty acids) of lavender extracts.

Fatty Acids	SCE (S40)	SCE (S45)	UAE
α-linolenic acid (18:3n-3)	31.67 ± 1.58 *	25.61 ± 1.25	11.82 ± 0.55
Palmitic acid (16:0)	49.80 ± 2.31	25.10 ± 0.87	18.71 ± 0.80
Linoleic acid (18:2n-6)	7.66 ± 1.02	16.81 ± 1.05	19.66 ± 0.85
Oleic acid (18:1n-9)	5.37 ± 0.89	11.35 ± 0.99	27.21 ± 1.10
Stearic acid (18:0)	1.55 ± 0.25	8.39 ± 0.11	13.73 ± 0.62
Behenic acid (22:0)	2.52 ± 0.12	5.79 ± 0.35	3.58 ± 0.20
Arachidic acid (20:0)	0.86 ± 0.16	5.63 ± 0.52	4.62 ± 0.25
Vaccenic acid (18:1n-7)	0.59 ± 0.11	1.31 ± 0.07	0.67 ± 0.08
SFAs	54.73 ± 1.25	44.91 ± 1.53	40.64 ± 1.35
MUFAs	5.93 ± 1.20	12.66 ± 1.28	27.88 ± 1.20
PUFAs	39.33 ± 1.48	42.43 ± 1.87	31.48 ± 1.35
*n-3* PUFAs	31.67 ± 1.59	25.61 ± 0.85	11.82 ± 0.55
*n-6* PUFAs	7.66 ± 1.58	16.81 ± 0.98	19.66 ± 0.90
*n-6*/*n-3*	0.24	0.66	1.66
PUFAs/SFAs	0.72	0.94	0.77

Abbreviations: SFAs—saturated fatty acids, MUFAs—monounsaturated fatty acids, PUFAs—polyunsaturated fatty acids. *—standard deviation.

**Table 3:** Microencapsulation efficiencies for selected phytochemical (%).

Selected Parameter	Variant 1 (CMC)	Variant 2 (AG)	Variant 3 (WPI-WPH)	Variant 4 (WPI-CN)
Total chlorophylls	41.97 ± 1.70 ^c,^*	49.02 ± 1.33 ^b^	95.22 ± 1.85 ^a^	94.65 ± 1.44 ^a^
Chlorophyll *a*	38.87 ± 1.43 ^c^	44.79 ± 0.57 ^b^	96.62 ± 2.87 ^a^	98.01 ± 1.56 ^a^
Chlorophyll *b*	43.90 ± 3.56 ^c^	51.79 ± 2.55 ^b^	90.61 ± 1.20 ^a^	90.61 ± 1.36 ^a^
Total carotenoids	49.32 ± 3.16 ^b^	50.14 ± 2.97 ^b^	94.79 ± 1.29 ^a^	97.04 ± 1.20 ^a^
Total polyphenols	61.79 ± 1.98 ^b^	61.92 ± 1.54 ^b^	93.88 ± 2.01 ^a^	93.93 ± 2.01 ^a^
Total flavonoids	73.36 ± 2.87 ^c^	69.82 ± 1.47 ^d^	90.22 ± 1.20 ^b^	90.72 ± 2.47 ^a^

Values are represented as mean ± standard errors (*). Superscript values that for the same column do not share the same letter (a, b, c and d) are significantly different at *p* < 0.05 based on the Tukey method. Different encapsulants were used, such as: Variant 1–agar and carboxymethyl cellulose, Variant 2–agar and acacia gum, Variant 3–agar and whey protein isolates and whey protein hydrolysates, Variant 4–agar and whey protein isolates and whey protein casein.

**Table 4:** Phytochemical profile of the powders.

Selected Parameter	Variant 1 (CMC)	Variant 2 (AG)	Variant 3 (WPI-WPH)	Variant 4 (WPI-CN)
Total chlorophylls (mg/g D.W.)	1.54 ± 0.05 ^a,^*	0.88 ± 0.03 ^b^	0.43 ± 0.05 ^c^	0.27 ± 1.44 ^d^
Chlorophyll *a* (mg/g D.W.)	0.61 ± 0.01 ^b^	0.68 ± 0.02 ^a^	0.17 ± 0.07 ^c^	0.10 ± 1.56 ^d^
Chlorophyll *b* (mg/g D.W.)	0.93 ± 0.10 ^b^	1.05 ± 0.10 ^a^	0.27 ± 0.02 ^c^	0.16 ± 1.36 ^d^
Total carotenoids (mg/g D.W.)	338.70 ± 24.60 ^a^	339.30 ± 2.39 ^a^	95.00 ± 1.29 ^b^	53.82 ± 1.20 ^c^
Total polyphenols (mg GAE/g D.W.)	5.17 ± 0.42 ^d^	5.47 ± 1.54 ^c^	9.71 ± 2.01 ^b^	10.78 ± 0.10 ^a^
Total flavonoids (mg CE/g D.W.)	4.25 ± 0.87 ^c^	3.98 ± 0.47 ^d^	6.61 ± 1.20 ^b^	6.92 ± 0.92 ^a^
Antioxidant activity (mMol Trolox/g D.W.)	2.85 ± 0.12 ^c^	3.44 ± 0.32 ^b^	4.77 ± 0.26 ^a^	4.80 ± 0.15 ^a^

Values are represented as mean ± standard errors (*). Superscript values that for the same column do not share the same letter (a, b, c and d) are significantly different at *p* < 0.05 based on the Tukey method.

**Table 5:** Particle characterization of the microencapsulated powders.

	Variant 1 (CMC)	Variant 2 (AG)	Variant 3 (WPH)	Variant 4 (WPI-CN)
D [2,3], µm	4.494	4.701	4.620	4.738
D [3,4], µm	4.684	5.048	4.909	5.108
D10, µm	3.613	3.648	3.635	3.654
D50, µm	4.497	4.608	4.563	4.629
D90, µm	5.698	7.612	6.665	7.860

D [2,3]–surface area mean; D [3,4]–volume moment mean; D10, D50 and D90–maximum diameter bellow which 10%, 50% and 90%, respectively, of the samples volume exist.

## Supplementary Materials

The following are available online. Figure S1: GC-MS chromatogram for the identification and quantification of volatile compounds from SCE (a) (S45–black, S40–pink) and UAE (b), Figure S2: GC-MS chromatogram for the identification and quantification of total fatty acids from SCE S40 (a) and S45 (b) fractions and UAE (c).
