# Supplementary material for: Fostering Lavender as a Source for Valuable Bioactives for Food and Pharmaceutical Applications through Extraction and Microencapsulation
Source: Molecules. 2020 Oct 28;25(21):5001. doi: 10.3390/molecules25215001 (PMC7662620; doi:10.3390/molecules25215001)
Supplement: Supplementary file 1 [file molecules-25-05001-s001.pdf]

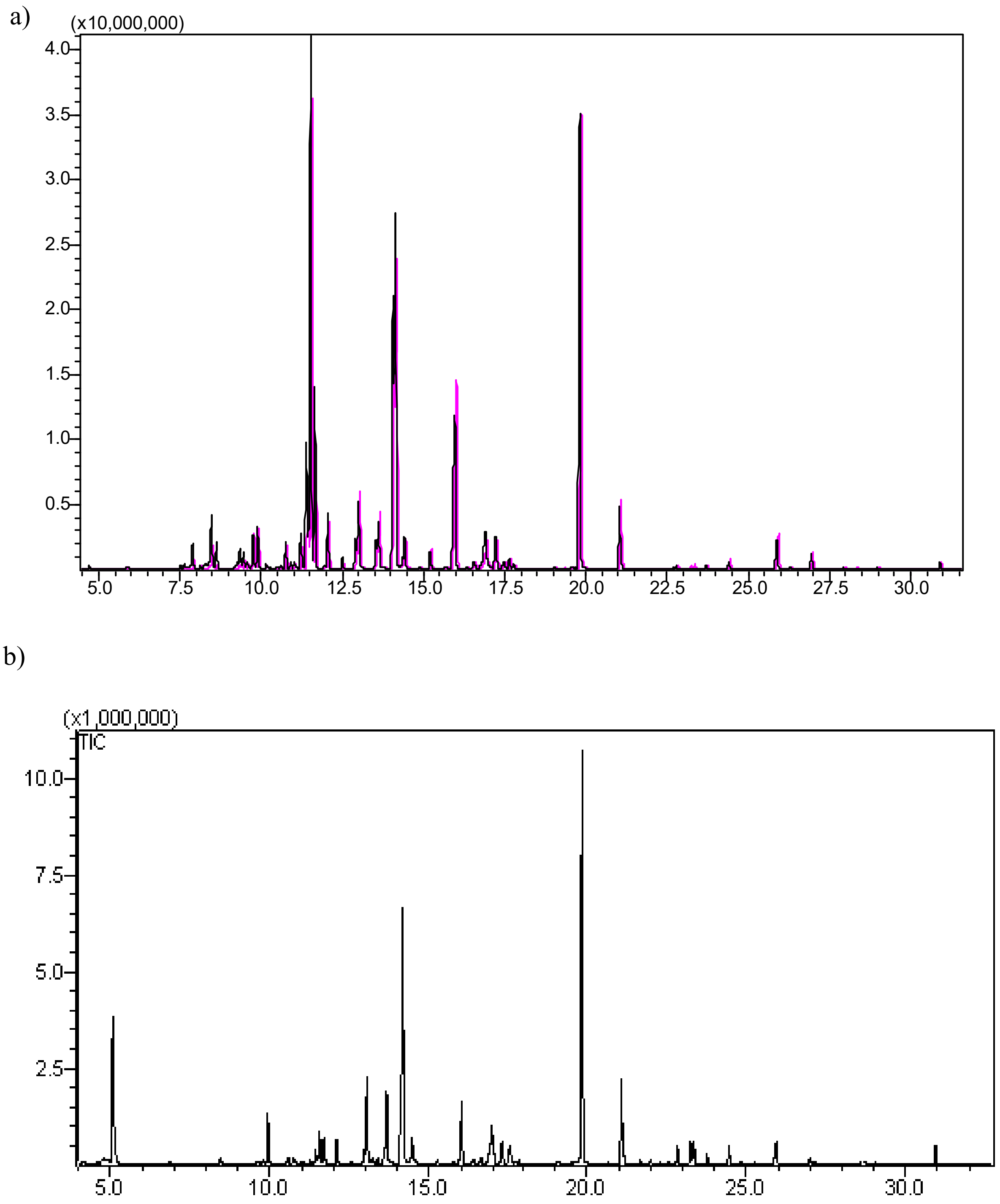

Figure 1. GC-MS chromatogram for the identification and quantification of volatile compounds from supercritical fluids extraction (SCE) (a) (S45— black, S40—pink) and ultrasound-assisted extraction (UAE) (b)

a)

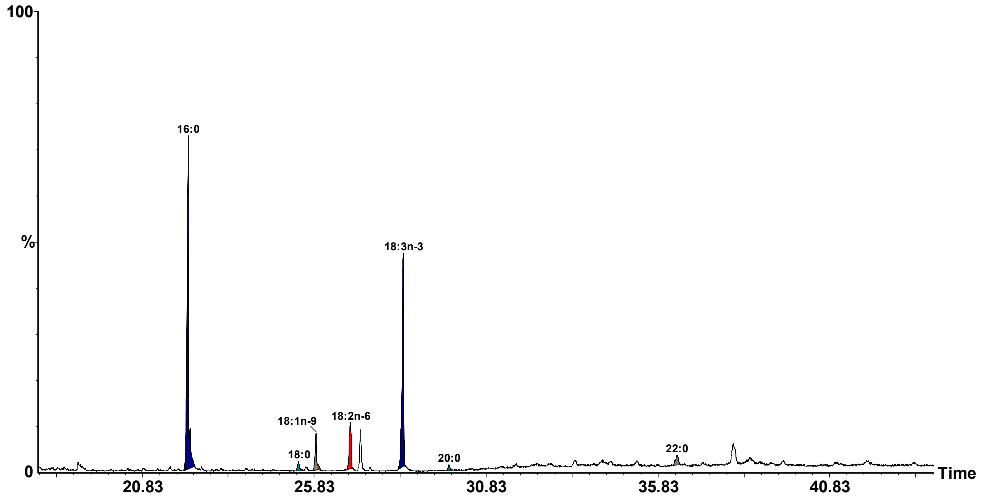
100
%
0
16:0
18:1n-9
18:0
18:2n-6
18:3n-3
20:0
22:0
20.83
25.83
30.83
35.83
40.83
Time

b)

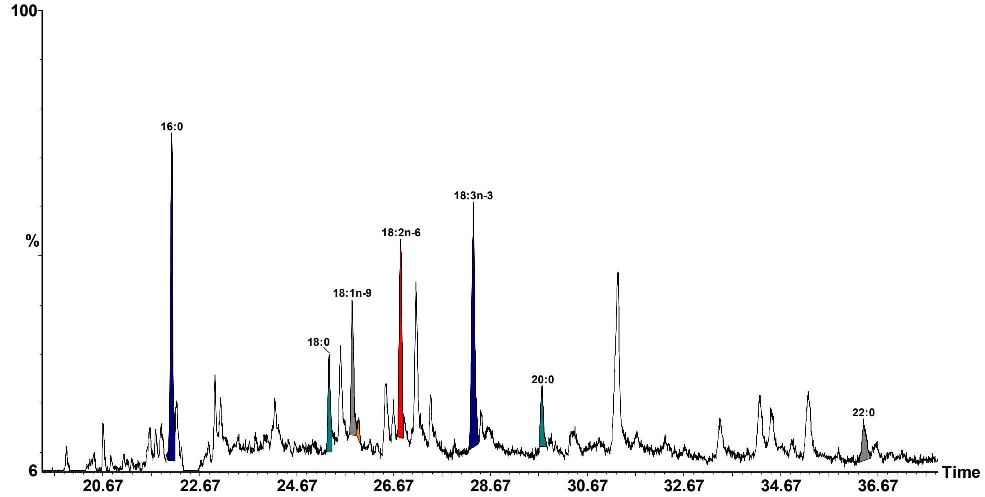
100
%
6
16:0
18:3n-3
18:2n-6
18:1n-9
18:0
20:0
22:0
20.67
22.67
24.67
26.67
28.67
30.67
32.67
34.67
36.67
Time

c)

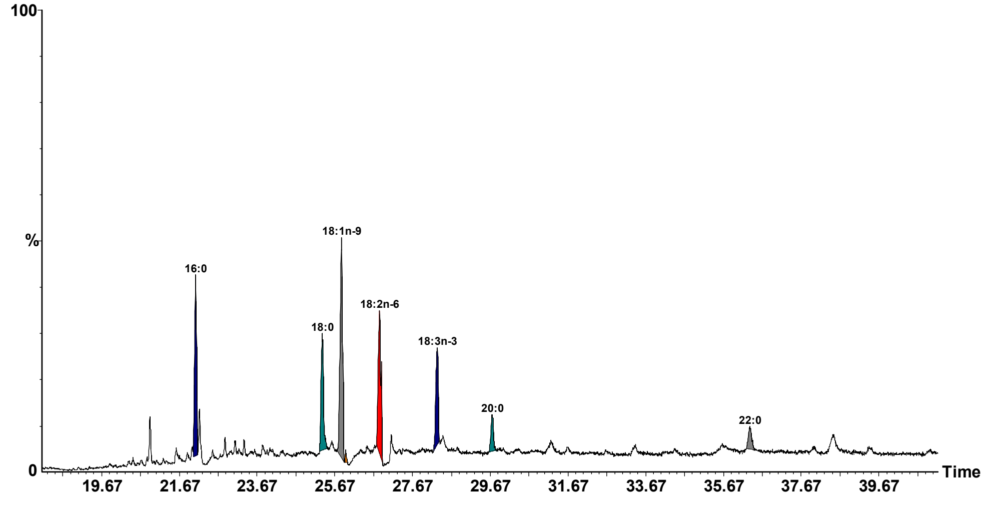
100
%
0
18:1n-9
16:0
18:2n-6
18:0
18:3n-3
20:0
22:0
19.67
21.67
23.67
25.67
27.67
29.67
31.67
33.67
35.67
37.67
39.67
Time

Figure 2. GC-MS chromatogram for the identification and quantification of total fatty acids from SCE S40 (a) and S45 (b) fractions and UAE (c)
